# Diagnosing Joubert Syndrome in Two Adult Siblings: A Very Rare Case Report

**DOI:** 10.7759/cureus.27042

**Published:** 2022-07-19

**Authors:** Pankaj K Kannauje, Vinay Pandit, Preetam Wasnik, Saroj K Pati, Nanditha Venkatesan

**Affiliations:** 1 General Medicine, All India Institute of Medical Sciences, Raipur, Raipur, IND; 2 Radiodiagnosis, All India Institute of Medical Sciences, Raipur, Raipur, IND; 3 Internal Medicine, All India Institute of Medical Sciences, Raipur, Raipur, IND

**Keywords:** joubert syndrome-related disorders (jsrds), molar tooth sign (mts), mri, hypotonia, joubert syndrome (js)

## Abstract

Joubert syndrome (JS) is a rare genetic disorder usually diagnosed during childhood. Adult Joubert syndrome is rare, and that too in siblings from a non-consanguineous marriage in their adulthood is extremely rare, with very few cases reported worldwide. The need for expensive imaging modality to aid diagnosis has also been cited as a drawback in diagnosing the condition in resource-poor areas. We describe the case of two adult siblings who came for other diseases and were diagnosed with Joubert syndrome.

## Introduction

Joubert syndrome (JS) is a rare disorder associated with malformations in the brainstem and cerebellum. Although the incidence of JS seems very low in our country, this could be heavily attributed to underreporting and lack of awareness of the condition [1]. In this article, we describe a case of JS, which was diagnosed in siblings from a non-consanguineous marriage in their adulthood, thus making it a very rare case.

## Case presentation

A 21-year-old male patient known to have hypothyroidism on levothyroxine was brought with a diagnosis of upper respiratory tract infection. On examination, the patient had slurred speech and a low intelligence quotient. On probing further, milestones were delayed, although there was no history suggestive of birth asphyxia. However, it was not of concern among family members since the rate of development was similar to the patient’s elder brother, now 24 years old. Interestingly, the patient’s elder brother also has a low intelligence quotient, speech difficulties, and poor adaptive skills. These siblings were a product of non-consanguineous marriage and had an uneventful birth history. Both brothers had appreciable dysmorphic facies, rounded eyebrows, lower lip eversion with trapezoid-shaped lips, and upturned noses.

Neurological examination revealed reduced power in both limbs and decreased tone suggestive of hypotonia. Cerebellar signs were present in the patient in the form of scanning speech, ataxia, horizontal nystagmus, pendular knee jerk, past pointing, rebound phenomenon, and intention tremor.

**Figure 1:**
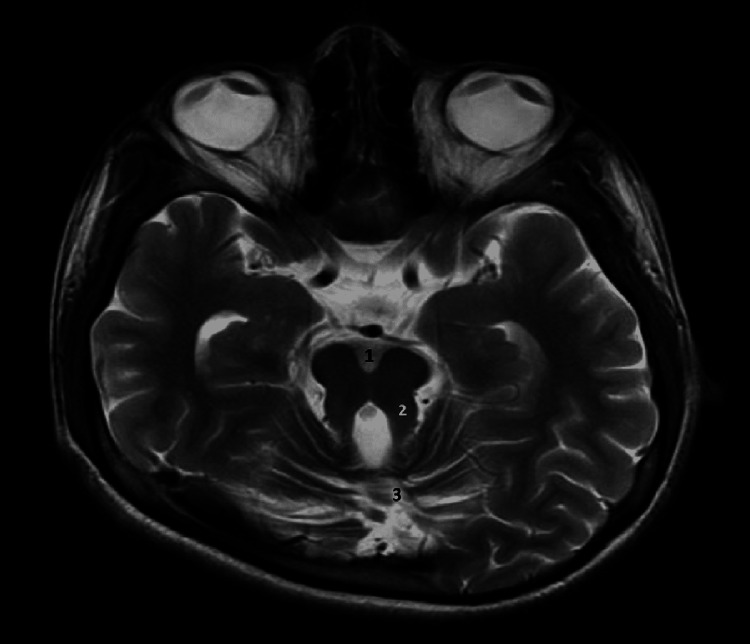
T2-weighted brain MRI in axial view showing deep interpeduncular fossa, elongated bilateral superior cerebellar peduncles, and hypoplasia of the cerebellar vermis, giving a molar tooth sign (MTS), consistent with Joubert syndrome

On neurological imaging, magnetic resonance imaging (MRI) of the brain T2-weighted images in axial view revealed features suggestive of Joubert syndrome (Figure 1).

**Figure 2:**
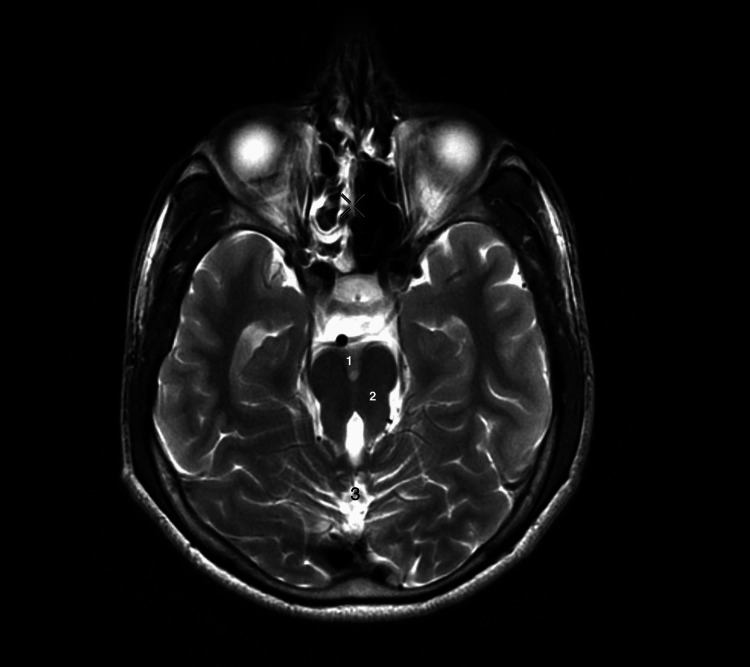
T2-weighted brain MRI in axial view of the sibling showing deep interpeduncular fossa, elongated bilateral superior cerebellar peduncle, and hypoplastic vermis consistent with Joubert syndrome

Following the diagnosis of Joubert syndrome in the patient, the relative brought the patient’s elder brother for evaluation. The brain MRI of the patient’s elder brother also revealed the same finding consistent with Joubert syndrome (Figure 2).

Apart from the thorough clinical examination, a complete workup was carried out in form of biochemical and radiological investigation to rule out any other organ involvement. A complete blood count revealed a mild microcytic hypochromic anemia. Elevated liver function enzymes and normal renal function tests were noted. Ophthalmological examination was within the normal limit. Ultrasonography of the abdomen revealed no abnormality in the liver and kidney. Given the absence of multi-organ involvement, the diagnosis of Joubert syndrome-related disorders (JSRDs) was excluded.

## Discussion

Of the 34 genes that cause JS, 33 are recessive, and one is X-linked [2]. Over the year, several genes were found to be causative of JS (e.g., 2q24.3, 2q13, 2q37.1, 3q11.1, 4p15.32, and 5p13.2), and this clearly justifies the phenotypic variability [3-5]. Consanguinity, as a plausible risk factor, was reported at a rate of 63.6% [6]. Recurrence risk among families has been estimated to be around 25% in the background of an autosomal recessive nature of inheritance, which would increase in cases with X-linked inheritance patterns.

Depending on the constellation of signs and symptoms seen in the patients, they can be diagnosed as a case of JS or Joubert syndrome-related disorders (JSRDs). The main neurological symptoms in JSRD are hypotonia, ataxia, developmental delay, intellectual disability, seizures, and abnormal ocular movements, and neurological symptoms are more consistently associated [7]. Dysmorphic facies include the presence of a large head with a prominent forehead, rounded eyebrows, epicanthal folds, ptosis, upturned nose with evident nostrils, open mouth, lower lip eversion in a trapezoid shape, and low-tilted ears [8].

Ophthalmological complaints comprise oculomotor apraxia, primary position nystagmus, strabismus or ptosis, pigmentary retinopathy, and decreased vestibulo-ocular reflexes. Nystagmus can be horizontal, vertical, and/or torsional and typically has a pendular or sometimes seesaw pattern [8]. Hepatic involvement can present not only with elevated liver function tests but also with portal hypertension, cirrhosis, hepatosplenomegaly, and esophageal varices [1].

Almost all children present with a delay in the acquisition of milestones and demonstrate intellectual disability of variable severity and across a variety of domains [3,4]. Studies have shown that expressive language is usually worse than receptive language resulting from oromotor apraxia.

Renal abnormalities include renal dysplasia, juvenile nephronophthisis, progressive interstitial fibrosis, and cystic dysplastic kidney. Among skeletal abnormalities, the most commonly reported abnormalities include postaxial polydactyly [7]. Evaluation of a child includes an MRI scan, retinal examination, renal ultrasonography, electroretinogram, and karyotyping [7]. Antenatal diagnosis in suspected patients has been aided by a fetal MRI scan between 20 and 22 weeks. There are various MRI features that are specific to Joubert syndrome (Table 1) [9,10].

**Table 1:** MRI features and signs specific to Joubert syndrome

Features and signs	
MRI features [9]	Absence of fiber decussation in superior cerebellar peduncles and cerebellar tracts
	Small dysplastic, hypoplastic, or aplastic cerebellar vermis with incomplete lobulation
	Abnormal inferior olivary nucleus
	Dysplasia and heterotopia of cerebellar nuclei
	Dysgenesis of the isthmus, which is seen as elongation and thinning of the post-mesencephalic junction and deep interpeduncular fossa
	Incomplete fusion of half of the vermis, creating a sagittal vermis cleft seen on axial or coronal MRI planes
Molar tooth sign [9]	Characterized by deepened interpeduncular fossa, hypoplasia of the vermis, and prominent thickened and elongated superior cerebellar peduncles
Batwing sign [10]	Hypogenesis of the vermis resulting in a triangular-shaped mid-fourth ventricle and a “batwing-shaped” (or umbrella sign) superior fourth ventricle, noted in the posterior fossa, attributed to dilatation
Buttock sign [10]	A sign formed due to the absence of the posterior vermian lobe, hence separating the cerebellar hemispheres by forming a cleft

Differential diagnoses can be broadly divided into two: MRI with molar tooth sign (MTS) and MRI without MTS. It is important to know that MTS is a nonspecific sign that is observed in cases such as cerebello-oculo-renal syndrome, cerebellar-vermis hypoplasia, Dekaban-Arima syndrome, Malta syndrome, Senior-Loken syndrome, and Bardet-Biedl syndrome [11]. In the abovementioned case, the diagnosis was made after establishing three cardinal diagnostic criteria, which include molar tooth sign (MTS), hypotonia in infancy with later ataxia, and developmental delays/intellectual disability [11].

## Conclusions

Prognosis largely depends on the variant of the disease and the extent, type, and severity of the involvement of the organs. Rationalized genetic testing according to genotype-phenotype correlations is needed. Developmental outcomes may vary in patients who died young, patients who survived with developmental delay, and patients whose developmental quotients fall within the mildly delayed range.

Management strategies are aimed at supportive and symptomatic treatment. Infant stimulation, rehabilitation therapy for cognitive difficulties, speech therapy, apnea monitoring, avoidance of drugs causing respiratory depression, special schooling, vocational training, and occupational therapy to help learn and work in a conducive environment are some multidimensional approaches to treat these patients. Multidisciplinary treatment, including genetic counseling and annual screening per diagnostic protocol, will aid in providing these patients with an improved quality of life and curb the occurrence of newer cases. Screening of siblings and relatives clinically and radiologically may increase awareness of the entity in the society and result in early diagnosis as well.
